# Retention of Urine, a Sequela of Scarlatina, Successfully Treated with Strychnia

**Published:** 1847-04

**Authors:** Geo. L. Upshur

**Affiliations:** Norfolk, (Va.,)


### *Retention of Urine, a Sequela of Scarlatina, successfully treated with Strychnia.* By Geo. L. Upshur, M. D.

W. H——, a delicate child, æt. 7, of a nervous temperament and strumous diathesis, was attacked January 21st with ordinary symptoms of catarrh, accompanied by considerable febrile excitement, though he did not feel sufficiently indisposed to go to bed. On the 22d, he complained a little of sore throat; had not been exposed to scarlatina, but there was a case of well marked rubeola two doors off. Fever continued moderate until the night of the 24th, when it became higher, and the catarrhal symptoms were entirely superseded by the sore throat. I was called to see him on the morning of the 25th, and found his condition as follows: Face flushed; throat red, swollen, and painful; headache; tongue furred white, with papillæ projecting; constant nausea and vomiting; and over the whole surface of the body there was an eruption partaking of the character of the rash of scarlatina and measles combined, but not sufficiently distinct to render the diagnosis certain; there was no cough or suffusion of the eyes. *Prescription.*—Warm mustard-pediluvium. ℞. Pulv. Hyd. Chlor. Mit. gr. viii, Pulv. Ipecac. gr. iv, M. Pil. 4.—S., one every two hours. 26th. Rash abundant, and evidently that of scarlatina; fever moderate; thirst; great tenderness of the surface where the eruption is found; bowels were moved during the night; no nausea; thirst less painful. The patient continued in this condition without other treatment than a dose of Ol. Ricini, until the 29th, when the throat being more painful, I prescribed an emetic of Ipecac. and a gargle made of infusion of carrots.

From this time the patient commenced to convalesce, and I should not have bestowed further attention upon the case, but for the illness of another child in the same house. On the 9th of February, he complained to his mother of pain about the neck of the bladder, and inability to urinate. I was requested to see him in the afternoon of the 10th. Found him feverish, the bladder considerably distended, and suffering with headache. Had passed little or no urine since early in the morning. Ordered him to be put in a warm bath, and to take an ounce of caster oil and forty drops of laudanum immediately.

11th. Bladder very much distended; passed a restless night; bowels were moved twice, but no urine came away during the night; this morning there is styllicidium. Introduced the catheter, the operation being somewhat difficult from the smallness of the parts, and the existence of a partial congenital phymosis. There were three spasmodic strictures, situated about half an inch apart, the first one being about an inch from the external orifice of the urethra. By keeping the point of the catheter steadily pressed upon the seats of stricture, in the course of ten minutes they gave way and the instrument entered the bladder. About eight ounces of urine were drawn off; when it ceased to flow, the catheter being clogged with mucous. After trying in vain to clear it with the wire staff, I was obliged to withdraw it, and he refused to permit me to introduce it again. The urine was turbid, and of the odour of asparagus.

12th. Passed no urine during the night, and has slept none; considerable fever and a haggard countenance. Ordered successively, a warm bath; tobacco leaf steeped in alcohol to the perineum; nauseating doses of tartar emetic: and last an opiate enema. All of these means failing to relieve, resorted again to the catheter, being obliged to have the patient forcibly held. Drew off about a quart of urine with entire relief.

13th. Symptoms as yesterday. Ordered a cathartic and large doses of the Tr. Ferri Chlor. Passed no urine during the day, and in the evening introduced the catheter.

14th. Could not get the catheter more than two inches in the urethra, the stricture refusing to yield though pressed upon for twenty minutes. Ordered forty drops of laudanum, and repeat it if necessary.

15th. Bladder much distended, although the urine has been dribbling away all night. Again tried the catheter. After holding it against the stricture a few minutes, the spasm suddenly gave way, and a stream of urine forcibly ejected the instrument from the urethra; he passed about half a pint, when it suddenly stopped, causing great pain. Ordered the following:

℞. Strychniæ, gr. ss.
Pulv. Rhei, gr. xx.
M. Pil. 8.—S. One every five hours.

16th. Has taken four pills, and passed during the night about half a pint of urine. Complains of pain in the epigastrium; there is some twitching of the extremities. Continue the pills every four hours. In the evening he was free from fever and had urinated tolerably freely during the day.

17th. Passes his urine better, but there is still some pain and spasm about the neck of the bladder. Continue treatment. *Evening*. Twitching better marked and very general; observed it even in the occipito-frontalis muscle. Urinates with little or no difficulty. Discontinue medicine.

18th. Bowels moved during the night, and urine voided as in health. From this time he convalesced rapidly, and is now (March 5th) perfectly well.

*Remarks.* No writer upon scarlatina speaks of retention of urine as one of its sequelæ, and I am disposed to look upon its occurrence in this instance as a coincidence instead of a sequence. The child was of a nervous, irritable temperament, and in all probability any febrile action continued for several days would have been followed by the same effect. Be this as it may, however, retention of urine, under any circumstances, may justly be considered a distressing and serious complaint, for the relief of which a variety of remedies have, from time to time, been suggested.

Mr. Holbrook (*Med. Chir. Rev., March*, 1824) recommends the use of active cathartics, and reports several cases of cure from their employment. This treatment is based upon the well-known fact that the rectum is seldom or never evacuated without a simultaneous emptying of the bladder.

M. Gerard (*Jour. des Connais. Med. Chir.*, *May*, 1835) successfully treated four cases of retention of urine by frictions with ointment of belladonna over the hypogastrium. Before resorting to this treatment, the warm bath, opiates, venesection, leeches and anodyne enemata were tried without success. The ointment was made by rubbing up ʒij. of the Ext. Belladon. with ℥j. of lard.

Dr. Somervail (*Am. Jour. Med. Sci.*, Vol. xvi, p. 250) relates a case cured by muriate of ammonia, in doses of five grains every hour. He thinks the retention was caused by a large dose of solution of morphia, which the patient had taken for the relief of violent pain.

The only case I can find recorded in which strychnia has been employed for retention of urine, is given by Dr. Cory (*Med. Chir. Rev.*, *July*, 1839.) The patient was left, after labour with her first child, with total loss of power on the part of the bladder. It was necessary, for her relief, to use the catheter twice a day for three weeks. She was ordered to take one-sixteenth of a grain of strychnia three times a day. "I was, I must confess," says the author," "somewhat surprised to find my patient, after taking the second dose, pass her urine *suâ sponte*, and with ease and comfort to herself." The medicine was continued until about a grain had been taken, when the cure was complete. The author does not mention the use of any other means except the catheter.

Ergot, muriated tincture of iron and cantharides, have been highly extolled in the treatment of retention of urine. I have never given any of these except the Tr. Chlor. Fer., which failed in the case above reported, although the trial was a fair one. I should be more disposed to rely, in future, upon the belladonna ointment or the strychnia. The efficacy of these remedies, however, depends, of course, upon the cause of the affection. In retention of urine, so often found in old men, and which is dependent upon enlargement of the prostate gland, I presume they would not benefit at all. But where the retention is dependent upon spasmodic stricture of the urethra, or paralysis of the fundus of the bladder, or upon both these conditions, I feel persuaded that but a very small proportion of such cases would refuse to yield to this treatment.

*Norfolk*, (*Va.*,) March 5th, 1847.

